# The Key Role of Glutathione Compared to Curcumin in the Management of Systemic Lupus Erythematosus: A Systematic Review

**DOI:** 10.7759/cureus.31324

**Published:** 2022-11-10

**Authors:** Niriksha Ravi, Silpa Choday, Vivig Shantha Kumar, Anil KC, Anusha Parisapogu, Blessing T Ojinna, Hadrian Hoang-Vu Tran, Mingma L Sherpa, Nilasma Shrestha, Lubna Mohammed

**Affiliations:** 1 Internal Medicine and Neurology, California Institute of Behavioral Neurosciences and Psychology, Fairfield, USA; 2 Internal Medicine, California Institute of Behavioral Neurosciences and Psychology, Fairfield, USA; 3 Internal Medicine/Family Medicine, California Institute of Behavioral Neurosciences and Psychology, Fairfield, USA; 4 Infectious Diseases, California Institute of Behavioral Neurosciences and Psychology, Fairfield, USA; 5 Internal Medicine/Neurology, California Institute of Behavioral Neurosciences and Psychology, Fairfield, USA; 6 Pathology and Internal Medicine, California Institute of Behavioral Neurosciences and Psychology, Fairfield, USA

**Keywords:** sle oxidative pathways, systemic lupus erythematosus, autoimmune disorders, new advances in sle, lupus treatment alternatives, glutathione and lupus, curcumin and lupus, sle pathogenesis, glutathione, curcumin

## Abstract

In recent years, many documented cases of systemic lupus erythematosus (SLE) have been on the rise. The complicated pathophysiology of the disease makes it challenging to manage. Two databases, PubMed and Google Scholar, have a detailed screening using keywords and Medical Subject Heading (MeSH) combinations. The words are "Systemic Lupus Erythematosus OR SLE OR Lupus," "Glutathione," and "Curcumin." Articles had a detailed process of screening and quality appraisal. Using the English language as a primary filtering parameter, papers over the last 20 years, dating from 2002 to 2022, are the basis of this review. We reviewed all possible human studies documenting the use of curcumin and glutathione for treating SLE. A total of 15 articles are part of this systematic review. Curcumin and glutathione can act as potent drugs for treating lupus. Curcumin can be a more promising alternative since it operates on various pathways and is a more easily accessible source.

## Introduction and background

Systemic lupus erythematosus (SLE) is a commonly seen autoimmune disorder with several relapses over time. Every year, there are about one to 10 new cases per million [1]. A 4:1 ratio in children and 9:1 in adults exist for lupus cases in females versus males. Females have a much higher incidence of this disease. Monozygotic twins compose 20%-30% of the caseload, thus showing a strong genetic role in the evolution of SLE [2]. Lupus usually affects many parts, such as the joints, skin, blood, kidneys, and heart, but it may risk affecting any other organ [3].

While the cause is unknown, its pathogenesis involves several factors. It may be genetic, drug-induced, or due to immune system modifications [1]. Over the years, despite extensive studies, the exact cause of lupus is not known, with several attempts to understand the mechanism and treat the condition using this knowledge [4]. Innate and adaptive immune systems both are involved in the development of lupus. A loss of self-tolerance plays a significant role in the development of SLE [5]. Reactive oxygen species and defective apoptosis mechanisms also trigger the innate and acquired immune systems [6]. The innate system has components such as Toll-like receptors (TLRs) that release interferons (IFNs), tumor necrosis factor (TNF), and interleukins (ILs), whereas the adaptive system flares up using the complement pathway and bone marrow and thymic type of lymphocytes, which plays a role in the pathophysiology of lupus [5]. During the disease, autoantibody formation against nuclear components leads to mild symptoms or severe multi-organ involvement, depending on the extent of the autoimmune responses [4].

The main goal of treating lupus is remission, with an added attempt to avoid any relapses [7]. Hydroxychloroquine, glucocorticoids, azathioprine, methotrexate, and mycophenolate mofetil are currently used [3,7,8]. Belimumab, rituximab, and cyclophosphamide are the newer drugs chosen nowadays [3]. For routine treatment, the drugs chosen are usually mycophenolate and hydroxychloroquine. Glucocorticoids and cyclophosphamide are opted for in severe cases due to their riskier side-effect profile. Immunosuppressants such as azathioprine are used if the above drugs fail. In instances where glucocorticoids show beneficial effects, an attempt to reduce the dose is equivalent to maintaining remission. Biologics (such as belimumab) can be used in non-reactive cases but shows variable responses. Rituximab shows not very satisfactory results and has a risk for progressive multifocal leukoencephalopathy in a few cases. Drugs such as ustekinumab and anifrolumab show positive results but have yet to get commercial usage approval [7].

Commonly used immunomodulators, glucocorticoids, and several other drugs might help with symptomatic management, but they blunt the ability to fight infections and malignancies. The side effects led to a need for further alternatives, perhaps a natural one, to treat autoimmune ailments [9]. A much more detailed understanding of the pathophysiology and how SLE occurs has led to the use of many more therapeutic options for managing SLE. In addition, many drugs in use cause adverse effects and increase morbidity in patients with severe comorbid disease. The need for alternative measures has led to a shift toward using natural drugs with lesser adverse effects as alternative options for treatment [8]. This systematic review provides an overview of the pathophysiology of SLE. In addition, it also helps compare two newer alternatives for treatment, namely, curcumin and glutathione.

## Review

Methods

Guidelines

Preferred Reporting Items for Systematic Reviews and Meta-Analyses (PRISMA) 2020 guidelines are the basis for formulating this review paper [10].

Search Databases

The databases used were PubMed, PubMed Central, Medical Literature Analysis and Retrieval System Online (MEDLINE), and Google Scholar.

Search Strategy

Screening is done from August 23 to August 31, 2022, with keywords, followed by a combination search of "Systemic Lupus Erythematosus OR SLE OR Lupus," "Glutathione," and "Curcumin" and Medical Subject Heading (MeSH) search with strategic use of terms with Boolean AND and OR to maximize search accuracy. We searched for two sets for both comparison groups. Table 1 provides an overview of all the articles narrowed to conduct the research.

**Table 1:** Overview of method section SLE: systemic lupus erythematosus; PMC: PubMed Central; MeSH: Medical Subject Heading; MEDLINE: Medical Literature Analysis and Retrieval System Online

Serial number	Keyword and MeSH combined (last four used to narrow articles)	Database	Studies
1	SLE/Lupus/systemic lupus erythematosus	PubMed/PMC/MEDLINE	160,804
2	SLE/Lupus/systemic lupus erythematosus	Google Scholar	672,000
3	Curcumin	PubMed/PMC/MEDLINE	32,744
4	Curcumin	Google Scholar	238,000
5	Glutathione	PubMed/PMC/MEDLINE	175,879
6	Glutathione	Google Scholar	1,080,000
7	SLE/Lupus/systemic lupus erythematosus and Glutathione	PubMed/PMC/MEDLINE	198
8	SLE/Lupus/systemic lupus erythematosus and Glutathione	Google Scholar	18,600
9	SLE/Lupus/systemic lupus erythematosus and Curcumin	PubMed/PMC/MEDLINE	42
10	SLE/Lupus/systemic lupus erythematosus and Curcumin	Google Scholar	9,530

MeSH terms (used in PubMed) used are as follows: ("Glutathione/administration and dosage" {Majr} OR "Glutathione/chemistry" {Majr} OR "Glutathione/drug effects" {Majr} OR "Glutathione/immunology" {Majr} OR "Glutathione/pharmacokinetics" {Majr} OR "Glutathione/pharmacology" {Majr} OR "Glutathione/therapeutic use" {Majr} OR "Glutathione/therapy" {Majr}) AND ("Lupus Erythematosus, Systemic/diet therapy" {Mesh} OR "Lupus Erythematosus, Systemic/drug therapy" {Mesh} OR "Lupus Erythematosus, Systemic/enzymology" {Mesh} OR "Lupus Erythematosus, Systemic/epidemiology" {Mesh} OR "Lupus Erythematosus, Systemic/etiology" {Mesh} OR "Lupus Erythematosus, Systemic/genetics" {Mesh} OR "Lupus Erythematosus, Systemic/immunology" {Mesh} OR "Lupus Erythematosus, Systemic/pathology" {Mesh} OR "Lupus Erythematosus, Systemic/physiology" {Mesh} OR "Lupus Erythematosus, Systemic/physiopathology" {Mesh} OR "Lupus Erythematosus, Systemic/prevention and control" {Mesh} OR "Lupus Erythematosus, Systemic/therapy" {Mesh}) and ("Lupus Erythematosus, Systemic/diet therapy" {Mesh} OR "Lupus Erythematosus, Systemic/drug therapy" {Mesh} OR "Lupus Erythematosus, Systemic/enzymology" {Mesh} OR "Lupus Erythematosus, Systemic/epidemiology" {Mesh} OR "Lupus Erythematosus, Systemic/etiology" {Mesh} OR "Lupus Erythematosus, Systemic/genetics" {Mesh} OR "Lupus Erythematosus, Systemic/immunology" {Mesh} OR "Lupus Erythematosus, Systemic/pathology" {Mesh} OR "Lupus Erythematosus, Systemic/physiology" {Mesh} OR "Lupus Erythematosus, Systemic/physiopathology" {Mesh} OR "Lupus Erythematosus, Systemic/prevention and control"(Mesh) OR "Lupus Erythematosus, Systemic/therapy"(Mesh) ) AND ( "Curcumin/administration and dosage" {Mesh} OR "Curcumin/chemistry" {Mesh} OR "Curcumin/organization and administration" {Mesh} OR "Curcumin/pharmacokinetics" {Mesh} OR "Curcumin/pharmacology" {Mesh} OR "Curcumin/physiology" {Mesh} OR "Curcumin/therapeutic use" {Mesh}).

Inclusion/Exclusion Criteria

Papers included in this study date from 2002 to 2022. The English language is a filter for selecting patients with SLE using either glutathione or curcumin. Only human studies are part of this study.

Quality Appraisal and Bias Assessment

The 15 chosen articles are then assessed using tools such as Scale for the Assessment of Narrative Review Articles (SANRA) and Assessment of Multiple Systematic Reviews (AMSTAR) checklists [11,12]. The cutoff included articles scoring 70% or more for inclusion in this systematic review. Table 2 summarizes this process.

**Table 2:** Quality assessment overview SANRA: Scale for the Assessment of Narrative Review Articles; AMSTAR: Assessment of Multiple Systematic Reviews

Tool	Description	Total score	Accepted score	Studies
SANRA	Six components: reasoning for the article's relevance, overview of substantial aims or questions, literature search details, accurate referencing, reasoning, and data representation	12	9	Beccastrini et al. [1], Rivas-Larrauri and Yamazaki-Nakashimada [2], Tsang-A-Sjoe and Bultink [3], Pan et al. [5], Shah et al. [6], Giraldo et al. [8], Catanzaro et al. [13], Connolly and Hakonarson [14], Nagafuchi et al. [15], Edwards et al. [16], Fu et al. [17], Tewthanom et al. [18], Rodrigues and Percival [19], and Mao et al. [20]
AMSTAR	Sixteen components: use of population, intervention, control, and outcome (PICO) criteria; deciding the review methods before conducting the review and reporting deviations; explanation of studies chosen for the review; use of exhaustive search strategy; duplicate search; removing duplicates; removing articles based on inclusion/exclusion criteria; description of studies chosen; note on bias in the study; funding sources; combination of results in a meta-analysis; impact of bias by studies in the meta-analysis; role of bias in results; variations in results; publication of bias assessment; and note on the conflict of interests	14	12	Zeng et al. [9]

Results

**Figure 1:**
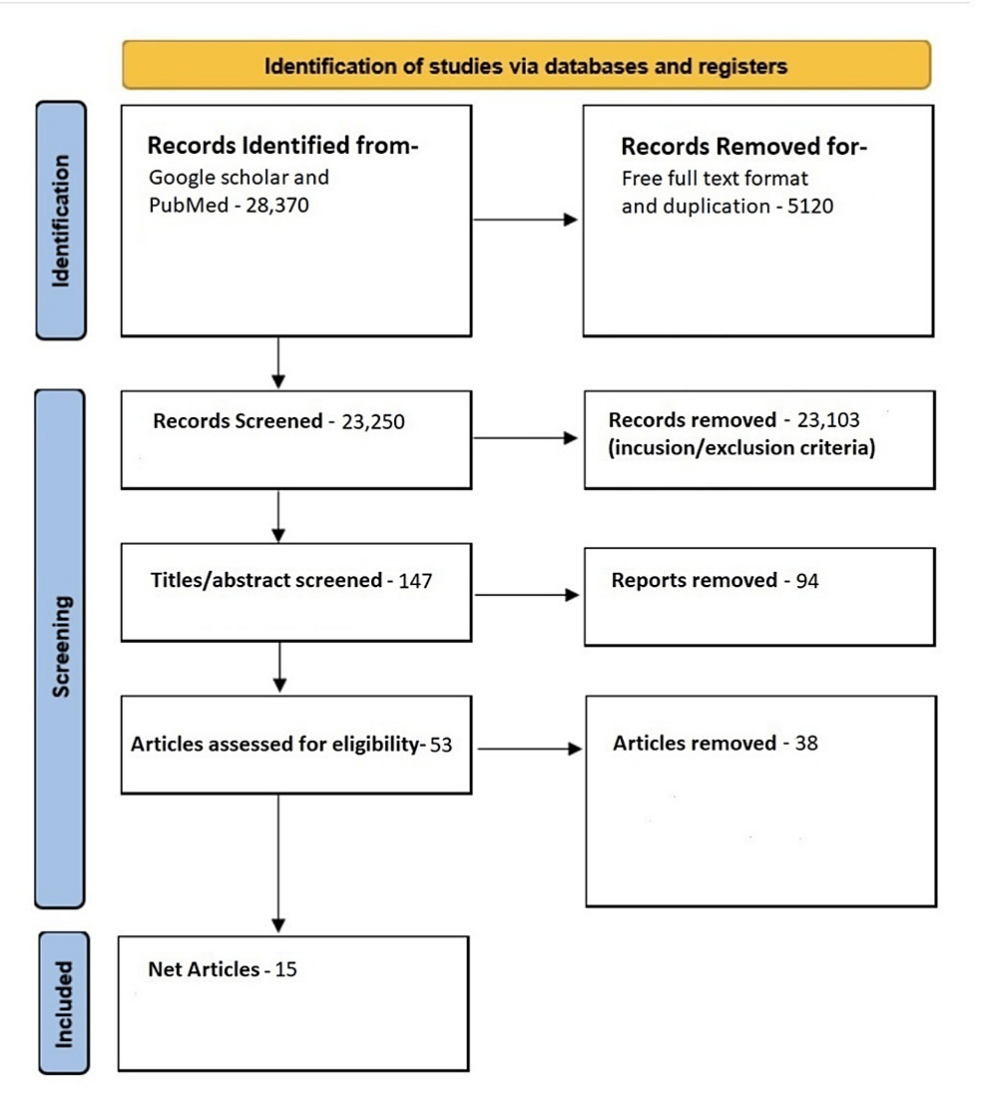
Preferred Reporting Items for Systematic Reviews and Meta-Analyses flow chart

All four databases undergo screening for groups with SLE using either curcumin or glutathione. Initially, we got 28,370 papers based on the search results. After applying several filters such as the English language, free flow text, and the inclusion/exclusion criteria, we came down to 147 papers. Later, we screened the titles and abstracts to narrow papers for quality appraisal and eligibility check. A total of 15 papers made it to the final review [1-3,5,6,8,9,13-20]. Figure 1 shows the process in detail.

Discussion

After noticing the widespread incidence of SLE and its multi-organ effects, we attempted to study the best possible treatment options. There are several gaps in what drug is best for managing patients regarding safety and efficacy. To study newer, less opted treatment drugs, we first reviewed the possible mechanisms of the emergence of SLE in great depth, following the study of the pathophysiology of SLE, by a parallel overview of the safer options, curcumin and glutathione, to deduce if they can play a significant role and which could be a better alternative drug.

Pathophysiology of systematic lupus erythematosus

The innate system consists of Toll-like receptors, complement pathways, and phagocytes. Innate cells use the human leukocyte antigen (HLA) and release several cytokines. These act like triggers for the acquired immunity cells to produce more inflammatory markers and autoantibodies. Acquired immunity, on the other hand, has memory and consists of T (thymic origin) and B (bone marrow-derived) lymphocytes. They work along with each other [13].

A theory suggests that the presence of interferons (IFNs) is the reason SLE happens. Type 1 IFN (alpha type) activates several cells such as lymphocytes and killer cells and reduces self-tolerance. Viral and bacterial deoxyribonucleic acid (DNA) are exogenous type 1 IFN inducers. They cause the nuclear factor kappa B (NFκB) pathway to activate and release IFN alpha, leading to autoantibody formation by B cells against the nuclear antigen of apoptosis cells. Reduced clearance of these apoptosis cells and debris acts as an endogenous type 1 IFN release trigger. The IFN flare-up causes a vicious cycle of autoimmune responses. In the innate system, the Toll-like receptors (TLRs) recognize accumulated apoptosis cells and cause a self-reaction. They also release interleukins (ILs) in response to the same [5]. Disruption in the IFN pathway and defective TLRs lead to SLE. Patients have increased TLRs on the surface of B cells, which undergo activation by self-antigens due to an impaired innate immune system [1]. The recognition of self-antigens by TLRs as foreign leads to a further increase in inflammatory response against the body [8].

Alterations in HLA and complement pathway deficiencies also play a crucial role in SLE development. Ninety percent of patients with a complement type 1 deficiency have developed SLE. Other deficiencies of complement types 2 and 4 also play a role in recurrent infections and the development of lupus [2]. It can be due to HLA mutations causing a complement type 1 (c1q type) deficiency, thus reducing complement types 2 and 4, and is responsible for SLE's evolution. Complement deficiency affects the clearance of compounds, leading to elevation in autoantigens and causing SLE. Even the defects of mannose-binding lecithin affect the complement pathway and act as a risk factor [1]. Complement defects are one of the major genetic risk factors causing lupus. Complement type 1 deficiency is pivotal in lupus development [14]. Sometimes, autoantibodies and self-antigens combine and get deposited as complement complexes. The interaction further triggers an immune response by causing a release of several interferons [8].

A new theory suggests that the excessive formation or reduced removal of structures called neutrophil extracellular traps (NETs) is responsible for autoimmune responses of SLE. NETs are composed of fibrous strands of nuclear and granular matter of neutrophils, along with proteins such as calprotectin, metalloproteinase, and lysosomal proteins. NETs trigger IFN synthesis and activate TLRs and inflammasomes to release ILs. NETs also play a role in reducing complement type 1, reducing the clearance of NETs and abnormal apoptosis cells [5].

Treatment drugs help reduce inflammatory markers and suppress autoimmune responses. Defective apoptosis leads to an increase in circulating self-antigens and triggers an autoimmune response [1]. An imbalance in the apoptosis rate and the accumulation of cellular debris lead to SLE. The debris causes an inflammatory response by activating nucleic acid recognition receptors, like TLRs. TLRs show linkage with type 1 IFN production. Type 1 IFNs (alpha and beta types) and other cytokines lead to B-cell differentiation and the formation of autoantibodies [15]. B cells not only produce our antibodies but also act as antigen-presenting cells and help aid the T-cell-mediated immune responses. Thus, reducing B-cell functioning can also help with lupus management [8].

Acquired immune system cells also contribute to SLE development. T and B cells also contribute to the pathogenesis of SLE. Some studies state that an excess of T helper cells in type 2 and a reduction in T helper cells in type 1 cause an increase in B-cell stimulation, thus leading to cell injury and autoantibody formation [5]. An increased expression of the killer-type T cells also leads to an induction of autoimmune responses [1]. In addition, a deficiency of regulatory T cells is one of the reasons SLE occurs in patients. They play a role in controlling inflammatory reactions and tolerance development [3]. A reduction or an absence of T regulatory cells significantly affects SLE evolution by autoimmune responses going unchecked [5].

There is a varied gene expression causing the disease to occur. In addition, modifying histone and DNA methylation can also lead to SLE [1]. Lastly, an interaction of genetic and environmental factors has led to the development of autoantibodies by triggering an autoimmune response [8]. Table 3 provides an overview of all the articles discussed in this section.

**Table 3:** Summary of articles used SLE: systemic lupus erythematosus

Reference number	Author, year, and type	Purpose	Results
[13]	Catanzaro et al., 2018, review article	Study the immune modulatory role of curcumin	Active components of medicinal plants can be used for treating several inflammatory autoimmune diseases in the long run
[5]	Pan et al., 2020, literature review	Explain the pathogenesis of SLE, and decide the treatment options based on the same	Understanding the mechanism of SLE evolution helps decide the target for drug action
[1]	Beccastrini et al., 2013, review article	Understand the immunopathogenesis of lupus, and understand the treatment options	Treatment can be tailored to various subsets of patients, depending on their preferences and response to treatment
[8]	Giraldo et al., 2012, review article	Analyze the mechanism of lupus and the molecules used for its management	Several alternative newer drugs can be used for patient management
[2]	Rivas-Larrauri and Yamazaki-Nakashimada, 2016, review article	Study various genetic defects associated with SLE to individualize treatment	Many genetic factors play a role in SLE development
[14]	Connolly and Hakonarson, 2012, literature review	Study genomic approaches toward SLE	Genetics can play a significant role in the development of lupus
[15]	Nagafuchi et al., 2019, review article	Discuss immune profiling of lupus and its role in medical management	Biomarkers will help guide the treatment and assessment of improvement
[3]	Tsang-A-Sjoe and Bultink, 2021, literature review	Compare newer drugs to decide on lupus treatment options	Newer treatment options are required due to toxic reactions of currently approved drugs

Curcumin target mechanism for SLE management

The ability of curcumin to interact with several immunomodulatory pathways makes it so valuable for managing autoimmune diseases [9]. The activation of oxidative pathways is how curcumin's anti-inflammatory role is activated [16]. It reduces the activation of the NFκB pathway and prevents IFN release by the innate system [9]. Curcumin targets the inhibitor of kappa-B kinase subunit beta (IKKβ) of the NFκB pathway to help reduce inflammation [16]. NFκB downregulation by curcumin minimizes the release of nitric oxide, ILs, and various other inflammatory markers from the T cells [13].

Curcumin also prevents neutrophil chemotaxis, thus reducing inflammatory responses in tissues. In addition, it helps regulate the deranged activity of TLRs against self-antigens [9]. Curcumin acts by causing impairment in the formation of pro-inflammatory markers [13]. Curcumin plays a role in adjusting the levels of inflammatory markers from cells. It reduced IL types 4, 6, and 8 and tumor necrosis factor (TNF) alpha. Reducing free radicals in the body helps reduce inflammatory changes in tissues [17].

It also helps regulate the stimulation of T helper cells, thus reducing the release of immune reactants. It also helped increase the T regulatory cells by reducing T helper 17 cells. This helped block the release of ILs, IFN, and TNF alpha [9]. Curcumin also suppresses the working membrane proteins responsible for T-cell functioning [13].

It also acts as a free radical scavenger and promotes glutathione synthesis. The potency of curcumin to inhibit the NFκB pathway depends on the cell's redox status. Glutathione protects cells from curcumin metabolites and limits their effects [17]. In addition, it also suppresses the Janus kinase/signal transducer and activator of transcription (JAK-STAT) pathway, thereby reducing a flared immune response. Curcumin thus reduces the expression of IL type 6 and TNF alpha [13]. Table 4 provides an overview of all the articles discussed in this section.

**Table 4:** Summary of articles used

Reference number	Author, year, and type	Purpose	Results
[9]	Zeng et al., 2022, systematic review	Study the efficacy and safety of curcumin in treating several autoimmune diseases	It shows promising results, but more studies need to be done for a stronger conclusive result
[16]	Edwards et al., 2017, review article	Study the role of curcumin metabolites	Curcumin inhibits several inflammatory markers and can be used as a modality for treating several diseases
[13]	Catanzaro et al., 2018, review article	Study the immune modulatory role of curcumin	Active components of medicinal plants can be used for treating several inflammatory autoimmune diseases in the long run
[17]	Fu et al., 2021, review article	Study the mechanism and medicinal uses of curcumin	Curcumin has a multipurpose role in immune modulation and can be used as a potent drug for treating several ailments in the future

Glutathione target mechanism for SLE management

SLE occurs due to an oxidative status imbalance. Studies show that a reduced glutathione level has a linkage with the occurrence of SLE [18]. Glutathione has antioxidant effects, and it also helps optimize the functioning of the lymphocytes. It protects the body cells from damage by reactive oxygen species. Glutathione sources are either food or direct supplementation [19].

Balancing levels of reactive oxygen species reduces the impact of inflammatory conditions by regulating the activation of NFκB and the oxidation of arachidonic acid [20]. Glutathione plays a role in preventing NFκB activation, thus reducing the release of several inflammatory compounds. It helps balance concentrations of ILs, IFNs, and TNFs [19]. Therefore, a glutathione deficit raises the risk for inflammatory diseases [20].

Studies show that glutathione replacement may help relieve complications of SLE. Glutathione reduces autoantibody levels in the body. It also helps regulate proper T-cell functioning [6]. A reduced level of glutathione in cases of SLE causes disrupted functioning of T regulatory cells [19].

The addition of many iron forms drives the cells toward ferroptosis. Ferroptosis has an iron-dependent accumulation of reactive oxygen species, depleting glutathione and causing lipid peroxidation and progression of ferroptosis [20]. It can also prevent cell damage by preventing the activation of the complement pathway, thus reducing damage by inflammatory cells [19].

Glutathione prevents excessive lipid peroxidation and oxidative stress in the body by maintaining the delicate balance of redox reactions [20]. Reactive oxygen species reduce the protective effects of glutathione from inflammatory mediators. Thus, the direct replacement of glutathione or the control of inflammatory mediators can help regulate the condition [6]. Table 5 provides an overview of all the articles discussed in this section.

**Table 5:** Summary of articles used

Reference number	Author, year, and type	Purpose	Results
[18]	Tewthanom et al., 2008, pilot study	Study the relation between glutathione and lupus severity	There is a relation between glutathione and lupus, but further studies are needed
[19]	Rodrigues and Percival, 2019, review article	Provide an overview for the roles of garlic, hydrogen sulfide, and glutathione in immune responses and treating diseases	Sulfur compounds play a significant role in inflammatory conditions and can be used for treatment
[20]	Mao et al., 2020, review article	Study the process of ferroptosis and its role in several diseases	Knowledge on ferroptosis may help in diagnosis and aid the treatment modalities for infections and inflammatory diseases
[6]	Shah et al., 2014, review article	Study ways of evolution of oxidative species and their role in diseases, along with the use of antioxidants	Oxidative biomarkers help guide the management of lupus

Limitations

This study has several limitations. The major one is the limited number of articles documenting the use of both compounds to treat SLE. More papers with interventions of curcumin and glutathione are needed to deduce its efficacy in a large pool of patients. Apart from this, the criteria set as filters for screening papers serve as a restriction. The use of only free full-text papers in the English language and including only human studies over the last 20 years do reduce the available resources.

## Conclusions

The available data acts as a basis to study SLE and its recent advances. An attempt at comparing two new alternatives helps us explore the possibility of treating SLE cases in the future. Detailed analysis and overview of the mechanism of evolution of SLE were of help to us in analyzing the two compounds based on their mechanisms of action. We also observed how both the innate and acquired immune systems are involved in the pathogenesis of lupus, thus allowing us to see the prospective scope for usage in SLE. Based on the data available, curcumin shows a broader range of applications. It has actions on many more pathways. Hence, curcumin is a more apt drug for usage soon. Curcumin is a more available, naturally occurring compound. In addition, some studies also indicate the effect of curcumin on glutathione function. Curcumin also has more studies proving its role in the management of SLE, thus making it a safer bet for case management.
